# High-density SNP-based linkage map construction and QTL analysis for growth-related traits in *Luciobarbus brachycephalus* using whole-genome resequencing data

**DOI:** 10.3389/fgene.2025.1644874

**Published:** 2025-08-18

**Authors:** Xuanpeng Wang, Dandan Gao, Gaowei Zhang, Yongchun Ge, Xinhai Wang

**Affiliations:** ^1^ Suqian Institute of Agricultural Sciences, Jiangsu Academy of Agricultural Sciences, Suqian, China; ^2^ Suqian King Crab Industry Research Institute, Suqian, China

**Keywords:** Luciobarbus brachycephalus, whole-genome resequencing, linkage map, QTL mapping, growth-related traits

## Abstract

**Introduction:**

*Luciobarbus brachycephalus* (commonly known as the Aral barbel) represents a commercially valuable fish species in China, contributing significantly to regional aquaculture economies. High-density genetic linkage mapping coupled with quantitative trait locus (QTL) analysis has emerged as a powerful approach for elucidating the genetic mechanism of complex traits in aquatic species.

**Method:**

The present study aimed to construct a SNP-based high-density linkage map using male parent, female parent, and 165 F_1_ full-sib progenies through whole-genome resequencing strategy, and subsequently perform comprehensive QTL mapping of six economically important growth-related traits, in order to identify candidate genes underlying growth regulation in *L. brachycephalus*.

**Results:**

Pearson correlation analysis demonstrated strong associations among all six growth-related traits (*r* > 0.8, *P* < 0.001), indicating likely pleiotropic regulation through shared genetic factors. The high-density linkage map for *L. brachycephalus* incorporated 164,435 high-quality SNPs distributed across 50 linkage groups, achieving complete genome coverage of 6,425.95 cM. The exceptional marker density (average inter-marker distance = 0.10 cM) establishes this as the most precise genetic map reported for this species to date, enabling the accurate candidate gene localization and enhanced marker-assisted selection. Through QTL mapping analysis, several genomic regions significantly associated with growth-related traits were identified based on genome-wide peak logarithm of odds scores. Specifically, one major QTL for body height was located on linkage group (LG27), and two distinct QTL for body weight were positioned on LG20 and LG26. Notably, four longitudinal growth traits (total length, body length, fork length, and preanal body length) were found to co-localize within the same significant QTL interval on LG27. These QTL intervals identified 6.27-39.36% of the phenotypic variance explained for the respective traits. Furthermore, putative candidate genes potentially regulating each target trait were identified through comprehensive analysis of these significant QTL intervals.

**Discussion:**

This integrated approach provides a foundation for marker-assisted selection and enhances the understanding of growth-related genetic mechanisms in this important species.

## Introduction

The Aral barbel, *Luciobarbus brachycephalus* (Cypriniformes, Cyprinidae, Barbinae), is native to the Aral Basin, Chu River drainage, and the southern and western Caspian Sea (Coad, 1998), and initially introduced to China from Uzbekistan’s Aral Sea in 2003 (Jiang et al., 2019). This species has become an economically significant aquaculture resource in China due to its rapid growth rate, palatability, and broad trophic adaptability. After 2 decades of domestication, annual production has reached approximately 10 million kilograms, generating an output value exceeding 400 million CNY (Geng et al., 2022). Notably, *L. brachycephalus* exhibits remarkable euryhaline tolerance, thriving in both freshwater and saline-alkaline aquatic systems (Geng et al., 2016), making it a promising candidate for aquaculture expansion in marginal environments. Consequently, *L*. *brachycephalus* has been successfully introduced to multiple regions across China and established as a commercially dominant aquaculture species. Its cultivation generates significantly economic, ecological, and social benefits for both farmers and consumers (Geng et al., 2012). However, the species faces a critical bottleneck restricted genetic diversity due to intensive inbreeding within small breeding populations. This genetic constraint stems from its recent introduction history and limited number of breeding stock. To support sustainable development of *L. brachycephalus* industry, there is an urgently need to develop improved *L. brachycephalus* varieties with enhanced growth performance and disease resistance through systematic breeding programs.

Growth traits represent critically important economic characteristics in aquaculture, receiving substantial focus in genetic improvement programs. Over recent decades, numerous improved aquatic animal varieties have been developed through conventional breeding approaches, including mass selection, family selection, and hybridization, with growth performance serving as the primary selection criterion. These traditional methods, which rely on phenotypic and pedigree evaluations at either individual or family levels, remain constrained by their labour-intensive nature and prolonged breeding cycles (Gjedrem, 2000; Rhode et al., 2023). In contrast, marker-assisted selection (MAS) offers a more precise and efficient alternative for genetic improvement. When validated molecular markers linked to target traits are available, MAS can significantly accelerate breeding progress by enabling early-life selection with greater accuracy (Tong and Sun, 2015).

The implementation of MAS in breeding programs requires prior identification of genomic regions and candidate genes associated with economically important traits. This foundational work enables precise selection of offspring carrying desirable genetic variants, thereby accelerating genetic improvement (Zhu S. R. et al., 2019). Quantitative trait locus (QTL) mapping has emerged as a powerful tool for elucidating the genetic architecture of complex traits in aquatic species (Ashton et al., 2017). Extensive QTL analyses have been conducted for various commercially relevant traits in aquatic animals, including sex determination (Loukovitis et al., 2012; Wang et al., 2015), disease resistance (Liu et al., 2016; Massault et al., 2011; Wang et al., 2017), stress resistance (Gu et al., 2018; Li et al., 2017; Moen et al., 2004), and body color (Jørgensen et al., 2018; Li et al., 2019). Particularly for growth-related traits (the most economically significant characteristics in aquaculture), QTL mapping has successfully identified regulatory genomic regions in multiple commercially important species, including *L*. *brachycephalus* (Geng et al., 2022), *Epinephelus lanceolatus* (Wu et al., 2023), *Argyrosomus japonicus* (Jackson and Rhode, 2024), *Argopecten scallops* (Wang F. K. et al., 2025), and *Acanthopagrus schlegelii* (Jia et al., 2025).

Early genetic mapping studies predominantly employed low-throughput molecular markers (e.g., random amplified polymorphic DNA, amplified fragment length polymorphism and simple sequence repeat) for linkage map construction, yielding limited marker density and consequently reducing QTL mapping resolution (Liu et al., 2012; Nomura et al., 2011; Zhang et al., 2010; Yin et al., 2022). The rapid development of next-generation sequencing (NGS) technologies has dramatically reduced genome sequencing costs (You et al., 2020), while enabling comprehensive detection of genetic variants from the whole genome-wide level, including single nucleotide polymorphisms (SNPs), indels, and structural variations (Reuter et al., 2015). This technological advancement has spurred the development of multiple high-throughput genotyping approaches, including restriction site-associated DNA sequencing (RAD-seq) (Chen et al., 2022), specific length amplified fragment sequencing (SLAF-seq) (Cui et al., 2021), 2b-RAD (Aslam et al., 2018), and whole genome resequencing (WGRS) (Mizukoshi et al., 2018). Among these, WGRS has emerged as a particularly powerful tool for population-scale variant discovery in both animals and plants (Lin et al., 2022; Yu et al., 2018), offering three key advantages for molecular breeding applications: (1) genome-wide characterization of genetic diversity (Wang X. et al., 2025; Wang S. L. et al., 2025), (2) construction of ultra-high-density linkage maps (Liu et al., 2022; Zhang et al., 2022), and (3) precise genome-wide association studies (Tang et al., 2023; Cui et al., 2025). Unlike reduced-representation methods, WGRS provides complete genomic coverage by sequencing the entire genome of organisms with established reference assemblies, thereby enabling exhaustive variant detection and functional annotation.

Current knowledge indicates that only one genetic linkage map exists for *L*. *brachycephalus*, developed using SLAF-seq strategy to facilitate growth-related QTL analysis. This map comprises 4,304 unique loci distributed across 50 linkage groups, spanning a total genetic distance of 2,419.2 cM (Geng et al., 2022). Genome-wide QTL analysis detected 72 statistically significant loci (LOD >3.0) associated with growth traits, distributed across nine linkage groups. These QTLs explained 9.2%–13.4% of the phenotypic variance (PVE) for the measured traits. Notably, no known growth-associated genes were found within the identified QTL regions. In the present study, we established an F1 full-sib family of *L. brachycephalus*, and employed WGRS to characterize genomic variations in the parental generation (male and female) and 165 progenies. This approach enabled the construction of a high-density SNP-based linkage map, and subsequently precise genome-wide QTL analysis to identify genomic regions and candidate genes associated with growth performance. These findings provide novel insights into the genetic mechanism of growth traits in *L. brachycephalus*, promoting the establishment of a framework for advanced genomic selection in this commercially important species.

## Materials and methods

### Ethics statement

All experimental procedures involving animal tissue collection were conducted in strict compliance with institutional ethical guidelines, and were approved by the Institutional Animal Care and Use Committee of the Suqian Institute of Agricultural Sciences, Jiangsu Academy of Agricultural Sciences (Approval No.20230714002, Suqian, China).

## Sample collection for linkage map construction

Parental stocks of *L*. *brachycephalus* exhibiting distinct phenotypic variants (yellow and red body coloration) were selected to generate an F1 full-sib mapping population. From this cohort, 200 progenies were randomly sampled for genetic analysis and maintained under controlled aquaculture conditions: water temperature 25.0 °C ± 0.5 °C, and dissolved oxygen >6.0 mg/L with continuous aeration. The fish were fed by a commercial diet (Runnong Animal Husbandry, Harbin, China) at a daily ration of 4 meals per day with each meal providing 2% of their body weight during the culture period. Following a 10-month growth period post-hatching, morphometric measurements were collected from the parental generation (male and female) and 165 offspring according to the Chinese National Standard GBT 18654.3–2008. Six economically significant growth traits were quantified using digital calipers (Deli, Zhejiang, China), including body weight (BW), total length (TL), body length (BL), body height (BH), preanal body length (PBL), and fork length (FL). Prior to tissue collection, specimens were anesthetized using buffered MS-222 (100 mg/L) until opercular movement ceased. White skeletal muscle samples were immediately excised, flash-frozen in liquid nitrogen, and stored at −80 °C to preserve genomic DNA integrity.

### WGRS and SNP calling

The genomic DNA was extracted from white muscle samples of the two parental individuals and 165 progenies using the Animal Genomic DNA Kit (Tiangen Biotech Co., Ltd., Beijing, China) following the manufacturer’s protocol. DNA quality was assessed through two complementary methods. First, DNA integrity was evaluated by electrophoresis on a 1.2% agarose gel, with visualization conducted using a ChemiDoc™ Touch gel imaging system (Bio-Rad, CA, United States). Subsequently, DNA concentration and purity were determined using a NanoDrop-2000 spectrophotometer (Thermo Fisher Scientific, MA, United States). Only DNA samples meeting the stringent quality criteria (*OD*_*260/280*_ ratio of 1.8–2.0 and *OD*_*260/230*_ ratio ≥2.0) were deemed suitable for sequencing library construction.

A total of 0.5 g of genomic DNA from each individual was used for library preparation. Sequencing libraries were constructed using the TruSeq Nano DNA HT Sample Prep Kit (Illumina, CA, United States) following the manufacturer’s protocol. The libraries were then sequenced on an Illumina NovaSeq X Plus system (Illumina, CA, United States) with a paired-end (PE) 350 bp strategy. Raw sequencing reads were subjected to quality filtering using Fastp (v0.20.0) (Chen et al., 2018) to remove low-quality reads (mean Phred score <20), including adapter-contaminated reads, and reads with excessive ambiguous bases (>10 Ns). The resulting high-quality clean reads were aligned to the *L. brachycephalus* reference genome (GWHBCHZ00000000, https://ngdc.cncb.ac.cn/gwh/) using BWA-MEME (v0.4.17) with default parameters (Jung and Han, 2022). The aligned reads were converted to BAM format, sorted using SAMtools (v1.11) (Li et al., 2009), and PCR duplicates were marked and removed with Picard (v3.0) (https://github.com/broadinstitute/picard, accessed on 17 July 2024). Variant calling was performed using GATK (v4.5.0.0) following the GATK Best Practices pipeline (McKenna et al., 2010), detecting SNPs and indels across all samples. The resulting variants were converted to PLINK format (PLINK v1.9 beta 7.7) and filtered to retain high-confidence SNPs by excluding loci with a call rate <80% across all samples, a minor allele frequency (MAF) < 0.01, monomorphic genotypes (only one allele present), and multiple SNPs within a single read (Purcell et al., 2007). Finally, the filtered SNPs were functionally annotated using snpEff (v4.3) (Cingolani et al., 2014) based on the reference genome annotation file. This pipeline ensured high-quality data for downstream genetic analyses.

### The construction of the high-density linkage map in *L*. *brachycephalus*

The linkage map of *L*. *brachycephalus* was constructed using JoinMap v5.0 (Ooijen and JoinMap, 2018) with SNPs classified into two segregation patterns: 1:2:1 (hk × hk, expected for heterozygous × heterozygous crosses) and 1:1 (nn × np or lm × ll, expected for heterozygous × homozygous crosses). A chi-square test (χ^2^, *P* < 0.001) was applied to assess deviations from expected Mendelian segregation ratios. Markers showing significant segregation distortion (*P* ≥ 0.05) were excluded to ensure map reliability. The remaining markers were grouped into 50 linkage groups at a logarithm of odds (LOD) threshold of 30, corresponding to the known chromosome number of *L. brachycephalus*. Lep-MAP3 was employed for regression mapping, with the OrderMarkers module optimizing marker positions within each LG. Recombination frequencies were converted to genetic distances (centiMorgans, cM) using Kosambi’s mapping function in Lep-map (Vinod, 2011). The final linkage map was visualized using the Perl SVG module, providing an intuitive graphical representation of marker distribution across linkage groups.

### Genetic correlation and QTL analysis of growth-related traits

Pearson’s correlation coefficients were calculated among the six economically important growth-related traits to evaluate their phenotypic relationships (Carr, 2012). Initial QTL mapping of growth-related traits was performed using Interval Mapping (IM) in MapQTL 6.0 software (Silva et al., 2012). Significance LOD thresholds of each phenotype were established through permutation tests (1,000 permutations, *P* < 0.05). The significant QTL intervals were assessed at both linkage group-wide and genome-wide levels using likelihood ratio statistics (1,000 replicates, 95% confidence). Growth-associated markers for each QTL were identified based on peak LOD positions and flanking regions using restricted Multiple QTL model (rMQM) analysis, which enhanced QTL detection accuracy while enabling identification of potential novel markers. The statistical significance of identified QTLs was determined using empirically derived LOD score thresholds. Only QTLs exceeding the permutation-derived LOD threshold (*P* < 0.05) were considered significant. Significant QTLs were graphically represented using MapChart 2.2 (Voorrips, 2002), displaying their genomic positions and effect sizes.

### Identification and characterization of candidate genes within significant QTL regions

Potential candidate genes regulating each trait were identified within QTL regions containing peak LOD scores. Growth-associated SNPs identified within QTL intervals were mapped to the *L. brachycephalus* reference genome. Protein-coding genes located within ±50 kb of significant SNPs were extracted from the reference annotation using BEDTools (v2.30.0), with genomic coordinates relative to the transcription start site.

## Results

### Characterization of the growth-related traits

**FIGURE 1:**
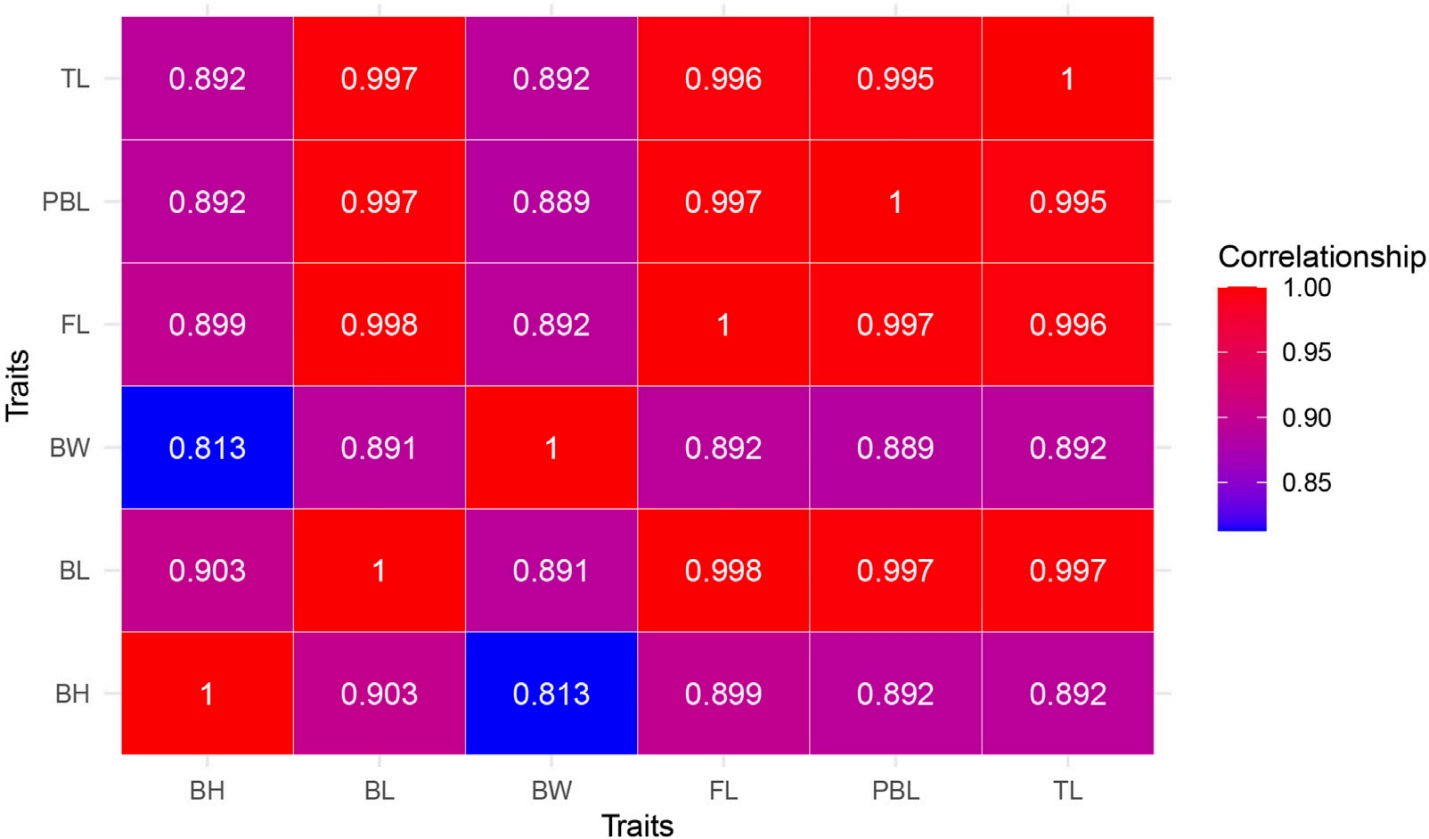
Heatmap visualization of Pearson correlation coefficients (*r*) among various growth-related phenotypic traits. The color gradient represents the strength and direction of pairwise correlations, with red hues indicating strong positive correlations (*r* = +1), green hues denoting weak or negative correlations (*r* = −1), and intermediate colors reflecting gradations in association strength. BW: body weight, TL: total length, BL: body length, BH: body height, PBL: preanal body length, FL: fork length.

The mean values (±SD) of growth-related traits among the 165 progenies were 15.34 ± 22.65 g for BW, 121.70 ± 41.34 cm for TL, 99.30 ± 34.31 cm for BL, 108.36 ± 36.31 cm for FL, 76.35 ± 24.67 cm for PBL, and 17.48 ± 6.90 cm for BH. The growth-related traits slightly deviate from a normal distribution, particularly in terms of BW (Supplementary Figure S1; Supplementary Table S1). Pearson correlation analysis was performed to assess pairwise relationships among six economically important growth-related traits in *L. brachycephalus* (Figure 1). All six growth-related traits showed strong positive correlations (*r* > 0.8). The strongest association occurred between BL and FL (*r* = 0.998, *P* < 0.05), while the weakest correlation was observed between BW and BH (*r* = 0.813, *P* < 0.05).

### Construction of sequencing libraries and sequencing

In the present study, WGRS was performed on both male and female parent, and 165 progenies using NGS technology. Sequencing generated approximate 72.04 million raw reads for the male parent and 57.85 million for the female parent. Additionally, progeny sequencing yielded approximate 28.75–46.81 million raw reads per individual, totalling 5.60 billion reads across all offspring. The combined sequencing output from both male and female parent and 165 progenies yielded 1,729.69 Gb of raw genomic data (Table 1).

**TABLE 1:** Summary of raw sequencing data.

Specimen	Raw reads	GC (%)	Q30 (%)
Male parent	72,042,403	37.81	94.63
Female parent	57,846,661	37.59	94.82
Maximum	46,813,309	38.15	95.24
Minimum	28,753,108	37.19	94.63
Total	57,275,460,063		
Average	67,440,617.6	37.72	94.95

Following quality filtering, mapping rates across progenies ranged from 99.59% to 99.79% (mean = 99.73%), demonstrating high sequencing accuracy in the present study. The draft genome of *L. brachycephalus* has been assembled, with a total size of 1,698.3 Mb (1.66 Gb) (Geng et al., 2021). The male and female parents yielded approximately 72 million and 58 million clean reads, achieving sequencing depths of 12.96× and 10.52×, respectively. Progeny sequencing yielded 28.42–46.28 million clean reads per individual, with an average depth of 5.99× across all samples (Table 2).

**TABLE 2:** Summary of clean data.

Specimen	Clean reads	GC (%)	Q30 (%)	Depth	Mapped ratio
Male parent	71,102,572	37.62	95.57	12.96	99.75
Female parent	57,235,215	37.47	95.55	10.52	99.72
Maximum	46,279,669	38.04	95.91	8.61	99.79
Minimum	28,424,424	37.09	95.42	5.47	99.59
Average	33,544,574.5	37.61	95.72	5.99	99.73

The present study finally identified 14,595,378 high-quality SNPs through comprehensive genome-wide analysis. Variant analysis identified approximately 7.88 million SNPs in the male parent (6,115,253 heterozygous and 1,764,021 homozygous) and 8.30 million SNPs in the female parent (6,592,962 heterozygous and 1,705,263 homozygous). Progeny SNP analysis revealed ranges of 6.53–7.88 million for total SNPs, 4.81–6.12 million for heterozygous sites, and 7.41–8.34 million for homozygous sites per individual (Table 3).

**TABLE 3:** Summary of SNPs from male parent, female parent, and progenies.

Specimen	SNP number	Ts/Tv	Heterozygosity	Homozygosity
Male parent	7,879,274	1.32	6,115,253	1,764,021
Female parent	8,298,198	1.32	6,592,962	1,705,263
Maximum	7,880,775	1.34	6,115,253	1,765,522
Minimum	6,525,981	1.31	4,806,857	1,719,124

### Construction of the high-density genetic linkage map

**FIGURE 2:**
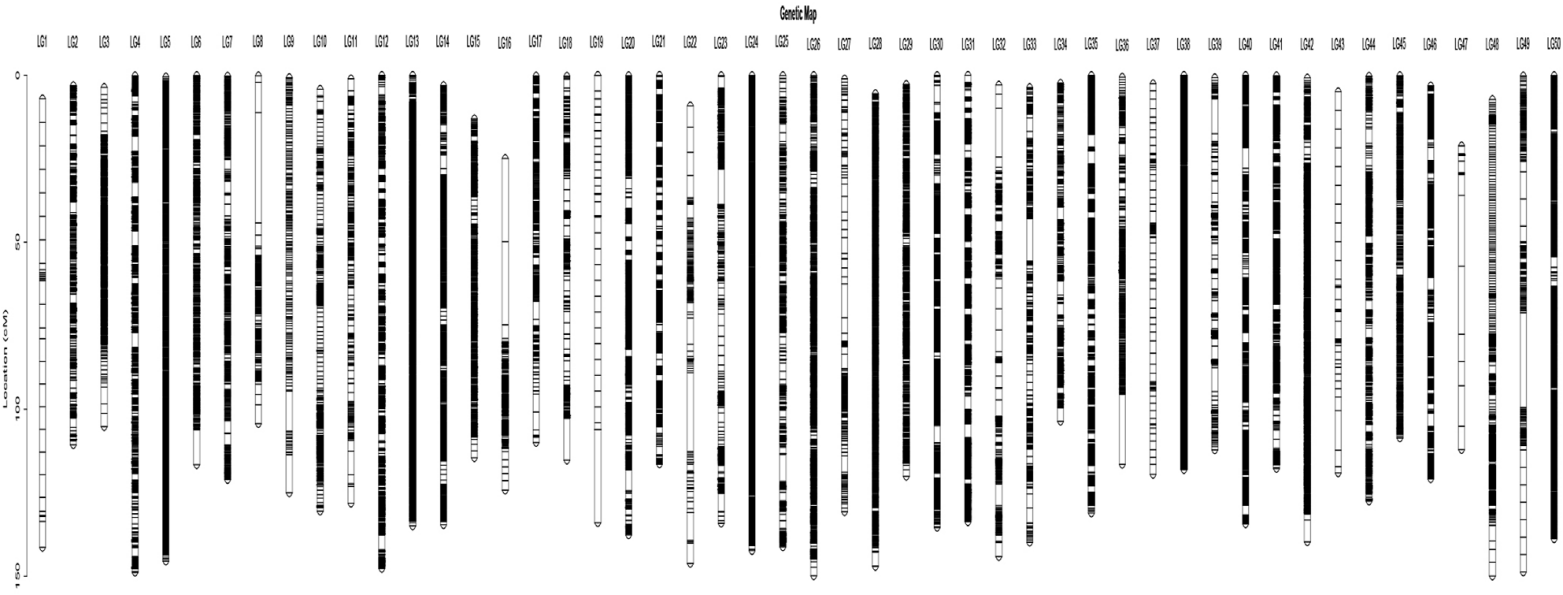
The consensus high-density SNP-based linkage map for *L*. *brachycephalus*.

The high-density genetic linkage map of *L. brachycephalus* contained 164,435 SNPs distributed across 50 linkage groups, spanning a total genetic distance of 6,425.95 cM (Table 4). Significant variation was observed in both SNP density and genetic distance across different linkage groups. The 50 linkage groups corresponded to the haploid chromosome number (n = 50) of *L. brachycephalus* (2n = 100), consistent with previous karyotypic reports (Figure 2) (Zhu C. et al., 2019). SNP distribution varied substantially across linkage groups, ranging from 221 (LG47) to 12,084 (LG38), with a mean density of 6,448.43 SNPs per linkage group. Genetic lengths varied among linkage groups, ranging from 103.61 cM (LG34) to 149.96 cM (LG48). The average inter-marker distance across linkage groups ranged from 0.01 cM (LG38) to 0.56 cM (LG9), with an overall mean of 0.10 cM. Gap analysis revealed that 99.62% of inter-marker intervals were <5 cM, with extreme values ranging from 0.41 cM (LG38) to 28.37 cM (LG49).

**TABLE 4:** Summary statistic of the linkage map of *L*. *brachycephalus*.

Linkage group	SNPs	Distance (cM)	Average (cM)	Max gap (cM)	Gap >5 cM (%)
LG1	257	141.38	0.55	7.74	0.07
LG2	3,699	110.64	0.03	3.19	0
LG3	3,622	105.23	0.03	4.97	0
LG4	8,095	148.82	0.02	4.52	0
LG5	6,269	145.49	0.02	0.67	0
LG6	1,001	116.63	0.12	10.40	0.01
LG7	779	121.08	0.16	3.63	0
LG8	5,371	104.31	0.02	3.05	0
LG9	223	125.05	0.56	12.01	0.01
LG10	4,708	130.53	0.03	2.60	0
LG11	1,845	128.16	0.07	7.08	0.01
LG12	7,061	147.70	0.02	4.50	0
LG13	9,726	134.96	0.01	0.99	0
LG14	2,196	134.61	0.06	4.03	0
LG15	1,190	114.54	0.1	2.07	0
LG16	2,845	124.25	0.04	2.91	0
LG17	4,941	109.97	0.02	5.38	0.01
LG18	1,721	115.25	0.07	12.64	0.02
LG19	780	133.99	0.17	27.84	0.03
LG20	1,741	137.63	0.08	6.21	0.01
LG21	5,654	116.39	0.02	2.55	0
LG22	873	146.17	0.17	23.59	0.01
LG23	3,355	134.16	0.04	1.57	0
LG24	7,109	142.36	0.02	1.12	0
LG25	4,203	141.23	0.03	8.18	0.01
LG26	2,854	149.89	0.05	2.56	0
LG27	529	130.71	0.25	10.18	0.01
LG28	1,301	147.10	0.11	4.16	0
LG29	1,411	120.11	0.09	1.97	0
LG30	3,741	135.38	0.04	4.92	0
LG31	6,637	133.72	0.02	4.08	0
LG32	1,794	144.14	0.08	14.66	0.02
LG33	1,100	139.84	0.13	12.70	0.02
LG34	4,421	103.61	0.02	4.20	0
LG35	5,616	131.11	0.02	3.84	0
LG36	760	116.53	0.15	20.92	0.03
LG37	1,489	119.52	0.08	3.72	0
LG38	12,084	118.09	0.01	0.41	0
LG39	1,980	112.18	0.06	10.41	0.02
LG40	3,577	134.34	0.04	6.17	0.01
LG41	6,317	117.75	0.02	2.72	0
LG42	3,059	139.71	0.05	6.52	0.01
LG43	791	119.09	0.15	11.87	0.02
LG44	3,693	127.53	0.03	4.35	0
LG45	669	108.62	0.16	2.16	0
LG46	4,496	120.83	0.03	3.97	0
LG47	221	112.09	0.51	21.23	0.03
LG48	2,874	149.96	0.05	4.62	0
LG49	581	148.76	0.26	28.37	0.02
LG50	3,266	138.82	0.04	3.13	0
Total	164,435	6,429.95			0.38
Average	6,448.43	128.59	0.10	8.37	

### Genome-wide QTL analysis reveals growth-related loci and candidate regulatory genes

**FIGURE 3:**
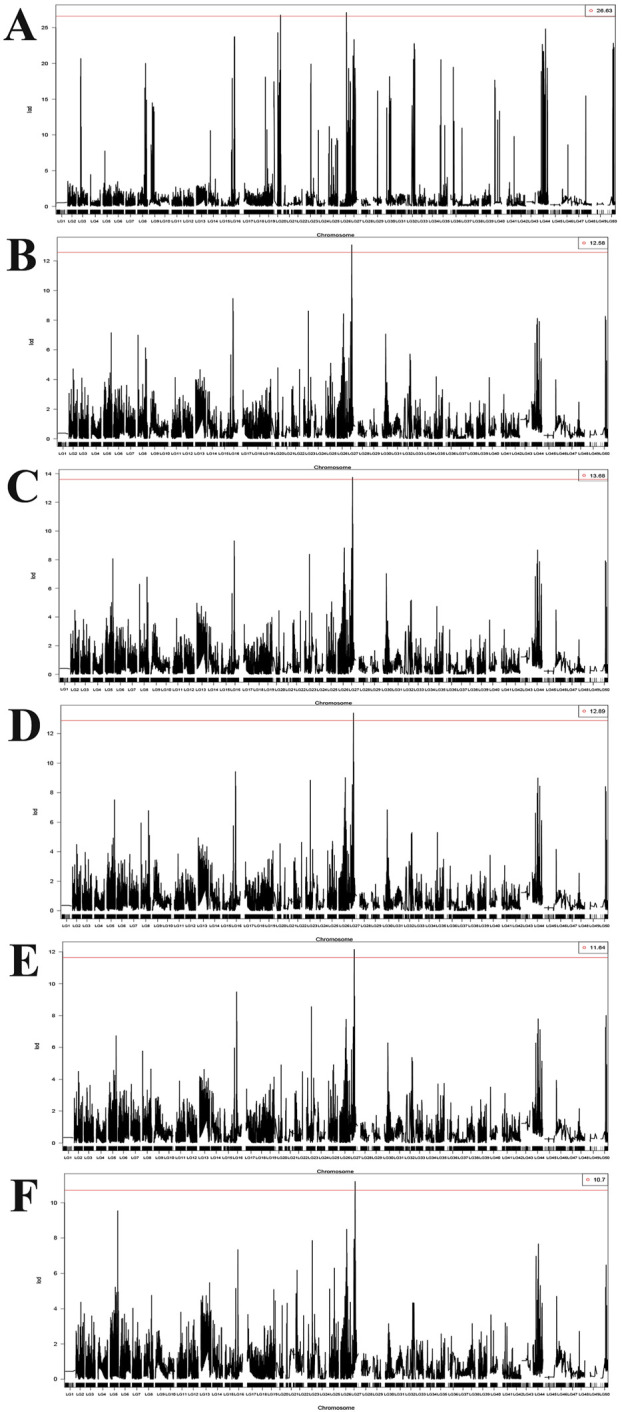
Genome-wide LOD score distributions for QTL analysis of **(A)** BW: body weight, **(B)** TL: total length, **(C)** BL: body length, **(D)** FL: fork length, **(E)** PBL: preanal body length, **(F)** BH: body height traits in *L*. *brachycephalus*. The plots depict LOD scores plotted against genetic linkage map positions, with horizontal red lines indicating genome-wide peak thresholds for significant QTL associated with each growth-related phenotype.

Genome-wide QTL mapping identified growth-related loci on three linkage groups: LG27 QTLs for BH, BL, TL, PBL, and FL, while BW QTLs were detected on LG20 and LG26 (Table 5; Figure 3). The BH-associated QTL on LG27 accounted for 6.27% of PVE with a peak LOD score of 11.20. Additionally, BW-associated QTLs on LG20 and LG26 explained 10.01% and 39.36% of PVE, with peak LOD scores of 27.13 and 26.77, respectively. Notably, BL, TL, PBL, and FL traits shared a common QTL interval on LG27, with respective PVE of 7.78% (LOD = 11.74), 7.49% (LOD = 13.08), 7.31% (LOD = 12.14), and 7.63% (LOD = 13.19).

**TABLE 5:** The information of growth-related QTLs for the six growth-related traits in *L. brachycephalus*.

Traits	LG	Locus	Peak LOD	PVE%	Gene	Mutation type
BH	27	2,916,912–2921,049	11.20	6.27	Histone deacetylase 1	Intron variation
BL	27	2,916,949–2921,032	13.74	7.78	Ethanolamine phosphotransferase 1	Intron variation
BW	20	2,363,237–5826,533	27.13	10.01	Nebulin-related-anchoring protein 1	Intergenic variation
					Myocyte-specific enhancer factor 2	
BW	26	2,474,271–2552,052	26.77	39.36	Serine/glucocorticoid-regulated kinase 1	Intergenic variation
					Cdc42-interacting protein 4	
TL	27	2,916,949–2921,032	13.08	7.49	Ethanolamine phosphotransferase 1	Intron variation
PBL	27	2,916,949–2921,032	12.14	7.31	Ethanolamine phosphotransferase 1	Intron variation
FL	27	2,916,949–2921,032	13.39	7.63	Ethanolamine phosphotransferase 1	Intron variation

Multiple candidate genes potentially regulating growth performance in *L. brachycephalus* were identified within the identified QTL intervals, located in close proximity to the peak marker positions (Table 5). Histone deacetylase 1 (*HDAC1*) was identified as a potential regulator of BH, while ethanolamine phosphotransferase 1 (*EPT1*) emerged as a candidate gene influencing multiple length traits (BL, TL, PBL, and FL). Additionally, four candidate genes were identified as potential regulators of BW, including nebulin-related anchoring protein 1 (*NRAP1*), myocyte enhancer factor 2 (*MEF2*), serum/glucocorticoid-regulated kinase 1 (*SGK1*), and Cdc42-interacting protein 4 (*CIP4*). The SNPs were located in intronic and intergenic regions of candidate regulatory genes, suggesting they do not directly alter protein-coding sequences but may influence gene regulation.

## Discussion

High-density linkage mapping and quantitative trait locus (QTL) analysis serve as powerful tools for identifying genomic regions and candidate genes underlying economically important traits in aquatic species. The present study aimed to construct a high-density genetic linkage map for *L. brachycephalus* using WGRS data, and perform comprehensive QTL mapping of six key growth-related traits. These efforts will facilitate the identification of functional genes regulating growth performance, providing novel insights into the molecular mechanisms controlling complex traits in this commercially valuable species.

Pearson’s correlation analysis has become a standard method for evaluating relationships between commercially important traits in aquaculture species. Following established threshold (*r* > 0.6 indicating strong correlations), numerous studies have documented high inter-trait correlations in various fish species (Mukaka, 2012). In *Epinephelus fuscoguttatus*, body weight and total length showed near-perfect correlation (*r* = 0.939), with 30 trait comparisons exceeding the *r* > 0.6 threshold (Zhang et al., 2022). *E*. *lanceolatus* exhibited strong correlations between body weight and total length (*r* = 0.82), body length (*r* = 0.80) and body height (*r* = 0.81) (Wu et al., 2023). *A*. *japonicus* demonstrated an exceptionally high weight-length correlation (*r* = 0.974) (Jackson and Rhode, 2024). The present study in *L. brachycephalus* revealed even stronger associations, with all six growth-related traits showing correlations >0.8 (*P* < 0.01). This remarkable synchrony in trait variation (Walsh and Blows, 2011) strongly suggests pleiotropic genetic regulation, where single genes may simultaneously influence multiple growth traits (Paaby and Rockman, 2013).

Prior to this study, only one high-density linkage map had been constructed for *L. brachycephalus*, utilizing the SLAF-seq approach (Geng et al., 2022). This earlier map comprised 4,304 unique SNP markers distributed across 50 linkage groups, spanning a total genetic distance of 2,419.2 cM with an average interval of 48.4 cM. In contrast, our study employed WGRS approach to construct a significantly enhanced linkage map. The resulting high-density map incorporates 164,435 SNP markers, maintaining the 50 linkage groups while achieving a substantially expanded total genetic length of 6,425.95 cM. Most notably, the average marker interval was reduced to just 0.10 cM, representing a 480-fold improvement in resolution compared to previous work. These dramatic improvements in both total map length and marker density provide valuable genetic resources for precise mapping of growth-related loci in *L. brachycephalus*, significantly advancing the potential for marker-assisted selection in this commercially important species.

QTL mapping represents a powerful methodological approach for elucidating the genetic mechanism underlying complex traits, particularly growth-related traits, across diverse fish species. The identification of key genes and functional polymorphisms associated with growth traits provides crucial insights into the molecular mechanisms governing fish growth and development. Currently, only a single published study has applied QTL mapping in *L. brachycephalus*, which successfully identified genetic loci influencing morphometric traits including body length, total length, and head breadth. Genome-wide QTL analysis identified 72 statistically significant loci (LOD >3.0) associated with growth traits, distributed across 9 distinct linkage groups. The PVE by these QTLs ranged from 9.2% to 13.4%. Notably, the prior investigation did not detect any growth-associated genes within the identified QTL regions, suggesting either limitations in genomic resources or the involvement of novel genetic factors (Geng et al., 2022). This limitation may be attributed to the relatively low marker density in the current linkage map. The current study detected growth-associated QTLs with LOD scores ranging from 11.20 to 27.13, explaining 6.27%–39.36% of the PVE. The LOD scores obtained in this study substantially exceeded those reported in previous QTL mapping studies of aquatic species. This increased detection power corresponds with the higher proportion of PVE, suggesting improved resolution of growth-related genetic traits (Sun et al., 2017; Wang et al., 2016). These results demonstrate significantly improved detection power and accuracy compared to previous QTL analyses of growth-related traits in aquatic animals. Notably, the QTL identified on LG27 exhibited strong associations with multiple growth-related traits, including BL, TL, PBL, and FL. This finding aligns with results from Pearson correlation analysis, which revealed near-perfect positive correlations (r ≈ 1) among these traits, suggesting a single gene may simultaneously influence multiple aspects of growth morphology.

The *EPT1* gene was localized within the significantly associated QTL interval for BL, TL, PBL, and FL. This positional and genetic association suggests *EPT1* may pleiotropically regulate these correlated growth traits. *EPT1*, which catalyzes the terminal step of the CDP-ethanolamine pathway, regulates growth in aquatic species by governing phosphatidylethanolamine (PE) biosynthesis (Vance, 2015). In teleosts, PE serves as both a structural membrane component and a signaling molecule. The cellular abundance of PE, critically regulates multiple fundamental growth processes, including membrane expansion during juvenile muscle development, hepatocyte proliferation, and rapid tissue growth phase (Hérault et al., 2010; Kwon et al., 2012; Hartmann et al., 2014).

The *HDAC1* gene was identified within the genetic region of QTL interval, highly associated with BH. *HDAC1* serves as a crucial epigenetic modulator that orchestrates growth regulation through transcriptional control of growth-associated genes, modulation of cellular proliferation rates, and regulation of differentiation pathways. Functioning as a lysine deacetylase, *HDAC1* catalyzes the removal of acetyl groups from histone tails, resulting in chromatin compaction and subsequent downregulation of key growth-related genes (Deng et al., 2021). While previous studies have established that *HDAC1* is essential for embryonic development (He et al., 2014) and cell cycle regulation (Vincent and Patrizia, 2006) in fish, our findings reveal a previously uncharacterized role for *HDAC1* in modulating skeletal growth. This study provides the first evidence linking *HDAC1* to osteogenic processes in *L. brachycephalus*, suggesting functional diversification of this epigenetic regulator in teleost.

The *NRAP1*, *MEF2*, *SGK1*, and *CIP4* genes were mapped within a significant QTL intervals linked to BW in *L. brachycephalus*, suggesting their potential functional roles in BW regulation. As a key structural regulator, nebulin critically controls thin filament length determination (Pappas et al., 2010). The C-terminal domain of nebulin is anchored within the Z-disk, while its N-terminal region extends toward the pointed end of thin filaments (Bang et al., 2002; Gokhin et al., 2012; Labeit and Kolmerer, 1995). Comparative studies reveal that nebulin-deficient skeletal muscle fibers exhibit significantly shorter thin filaments than wild-type counterparts, resulting in decreased thin-thick filament overlap and consequently impaired force generation capacity. Notably, emerging evidence indicates nebulin’s dual regulatory role in muscle contraction, where it modulates both cross-bridge cycling kinetics and enhances the calcium sensitivity of force production (Bang et al., 2009; Chandra et al., 2009; Ottenheijm et al., 2010). Protein kinases constitute a major class of regulatory enzymes in eukaryotic cells, modulating protein function and governing diverse cellular processes (Cipak, 2022). *SGK1*, a ubiquitously expressed serine/threonine kinase, plays a pivotal role in regulating ion channels, membrane transporters, transcription factors, and cellular enzymes (Waldegge et al., 1997; Webster et al., 1993), while also modulating neuronal excitability (Amato et al., 2009). Furthermore, SGK1 critically regulates fundamental cellular processes including growth, proliferation, survival, migration, and apoptosis (Lang et al., 2010; Amato et al., 2009; Bai et al., 2015; Schmidt et al., 2012). *MEF2* was initially observed in muscle, playing multiple roles in muscle cells to control myogenesis and morphogenesis (Black and Olson, 1998). *MEF2* family of transcription factors plays a central role in regulating muscle differentiation across animal species by controlling the expression of genes essential for myogenesis (Arredondo et al., 2001; Brunetti et al., 2015; Marín et al., 2004; Chechenova et al., 2015). In mammals, *MEF2* proteins coordinate multiple aspects of muscle development, including skeletal myofiber specification (Handschi et al., 2003; Liu et al., 2005) and differentiation (Black and Olson, 1998), cardiac development and hypertrophy (Lin et al., 1997; Moustafa et al., 2023), as well as vascular morphogenesis and smooth muscle proliferation (Bi et al., 1999; Zhao et al., 2002). For instance, *MEF2C* is indispensable for cardiac and smooth muscle differentiation during embryogenesis (Lin et al., 1997) and is required postnatally for skeletal muscle maintenance (Potthoff and Olson, 2007). *CIP4* functions as an effector of the small GTPase CDC42 (Aspenström, 2009) and localizes at cell-cell junctions, where it modulates cortical F-actin dynamics and the distribution of junctional proteins (Georgiou et al., 2008; Giuliani et al., 2009; Leibfried et al., 2008). *CIP4* plays an essential role in growth factor-mediated cell scattering by modulating cell-cell adhesion dynamics through regulation of junctional actomyosin contractility (Rolland et al., 2014).

## Conclusion

In conclusion, the present study constructed a high-density SNP-based genetic linkage map for *L. brachycephalus* using WGRS data. Analysis revealed strong correlations among six economically important growth-related traits (*r* > 0.8), suggesting pleiotropic regulation by shared genetic factors. A total of 146 million high-quality SNPs we identified from 165 F_1_ full-sib family progenies, of which 164,435 were successfully mapped. The final linkage map comprises 50 linkage groups spanning 6,425.95 cM, with an average genetic distance of 0.10 cM between markers, which is the highest density reported among aquatic species to date. Genome-wide QTL analysis identified multiple significant loci associated with growth-related traits, with peak LOD scores explaining 6.27%–39.36% of the PVE. Notably, a major QTL on LG27 showed strong pleiotropic effects, co-localizing with four morphometric traits, including BL, TL, PBL, and FL. This finding suggests shared genetic regulation underlying these correlated developmental traits. Using the Aral barbel genome, putative regulatory genes within QTL intervals were identified, including *HDAC1* on LG27 for BH, *EPT1* on LG27 for BL, TL, PBL, and FL, and *NRAP1* and *MEF2* on LG20, and *SGK1* and *CIP4* on LG26 for BW. This refined map enables precise localization of growth-related candidate genes, significantly advancing marker-assisted breeding for *L. brachycephalus*. The morphological data for the six growth-related traits exhibited slight deviations from a normal distribution, particularly BW. To enhance mapping accuracy, we plan to increase the number of sequenced individuals in future studies.

## Data Availability

The data presented in the study are deposited in the NCBI repository, accession number PRJNA1293864.

## Supplementary material

SUPPLEMENTARY FIGURE S1 The distribution of six growth-related traits. BW, body weight; TL, total length; BL, body length; FL, fork length; PBL, preanal body length; BH, body height traits.

SUPPLEMENTARY TABLE S1 Morphological measurements of six growth-related traits. BW, body weight; TL, total length; BL, body length; FL, fork length; PBL, preanal body length; BH, body height traits.

## References

[B1] Amato R. D’Antona L. Porciatti G. Agosti V. Menniti M. Rinaldo C. (2009). Sgk1 activates MDM2-dependent p53 degradation and affects cell proliferation, survival, and differentiation. J. Mol. Med. 87, 1221–1239. 10.1007/s00109-009-0525-5 19756449

[B2] Arredondo J. J. Ferreres R. M. Maroto M. Cripps R. M. Marco R. Bernstein S. I. (2001). Control of Drosophila paramyosin/miniparamyosin gene expression. Differential regulatory mechanisms for muscle-specific transcription. J. Biol. Chem. 276, 8278–8287. 10.1074/jbc.M009302200 11110792

[B3] Ashton D. T. Ritchie P. A. Wellenreuther M. (2017). Fifteen years of quantitative trait loci studies in fish: challenges and future directions. Mol. Ecol. 26, 1465–1476. 10.1111/mec.13965 28001319

[B4] Aslam M. L. Carraro R. Bestin A. Cariou S. Sonesson A. K. Bruant J. (2018). Genetics of resistance to photobacteriosis in gilthead sea bream (*Sparus aurata*) using 2b-RAD sequencing. BMC Genomics 19, 43–14. 10.1186/s12863-018-0631-x 29996763 PMC6042378

[B5] Aspenström P. (2009). Roles of F-BAR/PCH proteins in the regulation of membrane dynamics and actin reorganization. Int. Rev. Cell Mol. Biol. 272, 1–31. 10.1016/S1937-6448(08)01601-8 19121815

[B6] Bai J. A. Xu G. F. Yan L. J. Zeng W. W. Ji Q. Q. Wu J. D. (2015). SGK1 inhibits cellular apoptosis and promotes proliferation via the MEK/ERK/p53 pathway in colitis. World J. Gastroenterol. 21, 6180–6193. 10.3748/wjg.v21.i20.6180 26034353 PMC4445095

[B7] Bang M. L. Gregorio C. Labeit S. (2002). Molecular dissection of the interaction of desmin with the C-terminal region of nebulin. J. Struct. Biol. 137, 119–127. 10.1006/jsbi.2002.4457 12064939

[B8] Bang M. L. Caremani M. Brunello E. Littlefield R. Linari M. Chen J. (2009). Nebulin plays a direct role in promoting strong actin-myosin interactions. FASEB J. 23, 4117–4125. 10.1096/fj.09-137729 19679637 PMC2812046

[B9] Bi W. Drake C. J. Schwarz J. J. (1999). The transcription factor MEF2C-null mouse exhibits complex vascular malformations and reduced cardiac expression of angiopoietin 1 and VEGF. Dev. Biol. 211, 255–267. 10.1006/dbio.1999.9307 10395786

[B10] Black B. L. Olson E. N. (1998). Transcriptional control of muscle development by myocyte enhancer factor-2 (MEF2) proteins. Annu. Rev. Cell Dev. Biol. 14, 167–196. 10.1146/annurev.cellbio.14.1.167 9891782

[B11] Brunetti T. M. Fremin B. J. Cripps R. M. (2015). Identification of singles bar as a direct transcriptional target of *Drosophila* Myocyte enhancer factor-2 and a regulator of adult myoblast fusion. Dev. Biol. 401, 299–309. 10.1016/j.ydbio.2015.02.026 25797154 PMC4424145

[B12] Carr R. (2012). XLStatistics 12.11, 22. Australia: XLent Works.

[B13] Chandra M. Mamidi R. Ford S. Hidalgo C. Witt C. Ottenheijm C. (2009). Nebulin alters cross-bridge cycling kinetics and increases thin filament activation: a novel mechanism for increasing tension and reducing tension cost. J. Biol. Chem. 284, 30889–30896. 10.1074/jbc.M109.049718 19736309 PMC2781488

[B14] Chechenova M. B. Maes S. Cripps R. M. (2015). Expression of the troponin C at 41C gene in adult Drosophila Tubular muscles depends upon both positive and negative regulatory inputs. PLoS One 10, e0144615. 10.1371/journal.pone.0144615 26641463 PMC4671713

[B15] Chen S. Zhou Y. Chen Y. Gu J. F. (2018). fastp: an ultra-fast all-in-one FASTQ preprocessor. Bioinformatics 34, i884–i890. 10.1093/bioinformatics/bty560 30423086 PMC6129281

[B16] Chen B. Zhong P. Wu X. Peng K. Sun Y. Chen X. (2022). Construction of a genetic linkage map, QTLs mapping for low salinity and growth-related traits and identification of the candidate genes in Pacific white shrimp (*Litopenaeus vannamei*). Aquacult. Rep. 22, 100978. 10.1016/j.aqrep.2021.100978

[B17] Cingolani P. Platts A. Wang L. L. Coon M. Nguyen T. Wang L. (2014). A program for annotating and predicting the effects of single nucleotide polymorphisms, SnpEff: SNPs in the genome of *Drosophila melanogaster* strain w1118; iso-2; iso-3. SnpEff. Fly. 6, 80–92. 10.4161/fly.19695 PMC3679285 22728672

[B18] Cipak L. (2022). Protein kinases: function, substrates, and implication in diseases. Int. J. Mol. Sci. 23, 3560. 10.3390/ijms23073560 35408921 PMC8998185

[B19] Coad B. W. (1998). Systematic biodiversity in the freshwater fishes of Iran. Can. J. Physiol. Pharm. 65, 101–108. 10.1080/11250009809386802

[B20] Cui W. Guan M. Sadek M. A. Wu F. Wu Q. Tan H. (2021). Construction of a genetic linkage map and QTL mapping for sex indicate the putative genetic pattern of the F1 hybrid Scylla (Scylla serrata♀× S. paramamosain♂). Aquaculture 545, 737222. 10.1016/j.aquaculture.2021.737222

[B21] Cui W. Su F. Liu S. L. Zhang L. B. Yang H. S. Rasoamananto I. (2025). Identification of candidate genes and SNPs associated with growth traits by genome-wide association study (GWAS) in sea cucumber (*Apostichopus japonicus*). Aquaculture 605, 742512. 10.1016/j.aquaculture.2025.742512

[B22] Deng Y. Gao H. Wang H. Chen L. B. (2021). Snail/HDAC1/2 mediate skeletal growth retardation in fetuses caused by prenatal nicotine exposure. Toxicology 459, 152847. 10.1016/j.tox.2021.152847 34245815

[B23] Geng L. Lu C. Tong G. Li C. Xu W. (2012). Development and characterization of twenty microsatellite markers for the endangered fish *Luciobarbus capito* . Conserv. Genet. Resour. 4, 865–867. 10.1007/s12686-012-9660-3

[B24] Geng L. Tong G. Jiang H. Xu W. (2016). Effect of salinity and alkalinity on *LucioBarbus capito* Gill Na+/K+-ATPase enzyme activity, plasma ion concentration, and osmotic pressure. Biomed. Res. Int. 2016, 4605839–7. 10.1155/2016/4605839 27981049 PMC5131231

[B25] Geng L. W. Zou M. Jiang H. F. Meng M. H. Xu W. (2021). Draft genome assembly of the aral barbel *Luciobarbus brachycephalus* using PacBio sequencing. Genome Biol. Evol. 13 (7), evab131. 10.1093/gbe/evab131 34255058 PMC8489429

[B26] Geng L. W. Meng M. H. Xue S. Q. Lv X. N. Zou M. Jiang H. F. (2022). Construction of a high density genetic map and QTL analysis of morphological traits in Aral barbel *LucioBarbus brachycephalus* (Teleost: Cyprinidae). Aquacult. Rep. 27. 10.1016/j.aqrep.2022.101404

[B27] Georgiou M. Marinari E. Burden J. Baum B. (2008). Cdc42, Par6, and aPKC regulate Arp2/3-mediated endocytosis to control local adherens junction stability. Curr. Biol. 18, 1631–1638. 10.1016/j.cub.2008.09.029 18976918

[B28] Giuliani C. Troglio F. Bai Z. Patel F. B. Zucconi A. Malabarba M. G. (2009). Requirements for F-BAR proteins TOCA-1 and TOCA-2 in actin dynamics and membrane trafficking during *Caenorhabditis elegans* oocyte growth and embryonic epidermal morphogenesis. PLoS Genet. 5, e1000675. 10.1371/journal.pgen.1000675 19798448 PMC2744924

[B29] Gjedrem T. (2000). Genetic improvement of cold-water fish species. Aquac. Res. 31, 25–33. 10.1046/j.1365-2109.2000.00389.x

[B30] Gokhin D. S. Kim N. E. Lewis S. A. Hoenecke H. R. D'Lima D. D. Fowler V. M. (2012). Thin-filament length correlates with fiber type in human skeletal muscle. Am. J. Physiol. Cell Physiol. 302, C555–C565. 10.1152/ajpcell.00299.2011 22075691 PMC3287155

[B31] Gu X. H. Jiang D. L. Huang Y. Li B. J. Chen C. H. Lin H. R. (2018). Identifying a major QTL associated with salinity tolerance in Nile Tilapia using QTL-Seq. Mar. Biotechnol. 20, 98–107. 10.1007/s10126-017-9790-4 29318417

[B32] Handschin C. Rhee J. Lin J. Tarr P. T. Spiegelman B. M. (2003). An autoregulatory loop controls peroxisome proliferator-activated receptor gamma coactivator 1alpha expression in muscle. Proc. Natl. Acad. Sci. U. S. A. 100, 7111–7116. 10.1073/pnas.1232352100 12764228 PMC165838

[B33] Hartmann A. Hellmund M. Lucius R. Voelker R. D. Gupta N. (2014). Phosphatidylethanolamine synthesis in the parasite mitochondrion is required for efficient growth but dispensable for survival of toxoplasma gondii. J. Biol. Chem. 289, 6809–6824. 10.1074/jbc.M113.509406 24429285 PMC3945342

[B34] He Y. Wu J. Mei H. Yu H. Sun S. Shou J. (2014). Histone deacetylase activity is required for embryonic posterior lateral line development. Cell Prolif. 47, 91–104. 10.1111/cpr.12081 24267956 PMC6496587

[B35] Hérault F. Saez G. Robert E. Mohammad A. A. Davail S. Chartrin P. (2010). Liver gene expression in relation to hepatic steatosis and lipid secretion in two duck species. Anim. Genet. 41, 12–20. 10.1111/j.1365-2052.2009.01959.x 19781035

[B36] Jackson T. K. Rhode C. (2024). A high-density genetic linkage map and QTL identification for growth traits in dusky kob (*Argyrosomus japonicus*). Aquaculture 586, 740786. 10.1016/j.aquaculture.2024.740786

[B37] Jia C. F. Meng Q. Chen S. Y. Sun R. J. Xu D. F. Zhu F. (2025). Construction of the first high-density genetic linkage map and QTL mapping for growth traits in black seabream (*Acanthopagrus schlegelii*). Aquaculture 595, 741588. 10.1016/j.aquaculture.2024.741588

[B38] Jiang H. Geng L. Yang J. Tong G. Li C. Xu W. (2019). The complete mitochondrial genome of the aral barbel *lucioBarbus brachycephalus* (cypriniformes: Cyprinidae: Barbinae). Mitochondrial DNA B 4, 3685–3686. 10.1080/23802359.2019.1678439 PMC7707559 33366143

[B39] Jørgensen K. M. Solberg M. F. Besnier F. Thorsen A. Fjelldal P. G. Skaala Ø. (2018). Judging a salmon by its spots: environmental variation is the primary determinant of spot patterns in *Salmo salar* . BMC Ecol. 18, 14. 10.1186/s12898-018-0170-3 29650003 PMC5897946

[B40] Jung Y. Han D. (2022). BWA-MEME: BWA-MEM emulated with a machine learning approach. Bioinformatics 38, 2404–2413. 10.1093/bioinformatics/btac137 35253835

[B41] Kwon Y. Yu S. I. Lee H. Yim J. H. Zhu J. K. Lee B. H. (2012). Arabidopsis serine Decarboxylase Mutants Implicate the roles of ethanolamine in plant growth and development. Int. J. Mol. Sci. 13, p3176–p3188. 10.3390/ijms13033176 PMC3317708 22489147

[B42] Labeit S. Kolmerer B. (1995). The complete primary structure of human nebulin and its correlation to muscle structure. J. Mol. Biol. 248, 308–315. 10.1016/s0022-2836(95)80052-2 7739042

[B43] Lang F. Perrotti N. Stournaras C. (2010). Colorectal carcinoma cells-regulation of survival and growth by SGK1. Int. J. Biochem. Cell Biol. 42, 1571–1575. 10.1016/j.biocel.2010.05.016 20541034

[B44] Leibfried A. Fricke R. Morgan M. J. Bogdan S. Bellaiche Y. (2008). Drosophila Cip4 and WASp define a branch of the Cdc42-Par6-aPKC pathway regulating E-cadherin endocytosis. Curr. Biol. 18, 1639–1648. 10.1016/j.cub.2008.09.063 18976911

[B45] Li H. Handsaker B. Wysoker A. Fennell T. Ruan J. Homer N. (2009). Genome Project Data Processing, the sequence alignment/map format and SAMtools. Bioinformatics 25, 2078–2079. 10.1093/bioinformatics/btp352 19505943 PMC2723002

[B46] Li H. L. Gu X. H. Li B. J. Chen C. H. Lin H. R. Xia J. H. (2017). Genome-wide QTL analysis identified significant associations between hypoxia tolerance and mutations in the GPR132 and ABCG4 genes in Nile Tilapia. Mar. Biotechnol. 19, 441–453. 10.1007/s10126-017-9762-8 28698960

[B47] Li B. J. Zhu Z. X. Gu X. H. Lin H. R. Xia J. H. (2019). QTL mapping for red blotches in Malaysia red Tilapia (Oreochromis spp.). Mar. Biotechnol. 21, 384–395. 10.1007/s10126-019-09888-9 30863905

[B48] Lin Q. Schwarz J. Bucana C. Olson E. N. (1997). Control of mouse cardiac morphogenesis and myogenesis by transcription factor MEF2C. Science 276, 1404–1407. 10.1126/science.276.5317.1404 9162005 PMC4437729

[B49] Lin Y. Zhou S. Liang X. Guo B. Han B. Han H. (2022). Chromosomal mapping of a locus associated with adult-stage resistance to powdery mildew from Agropyron cristatum chromosome 6PL in wheat. Theor. Appl. Genet. 135, 2861–2873. 10.1007/s00122-022-04155-3 35819492

[B50] Liu Y. Shen T. Randall W. R. Schneider M. F. (2005). Signaling pathways in activity-dependent fiber type plasticity in adult skeletal muscle. J. Muscle Res. Cell Motil. 26, 13–21. 10.1007/s10974-005-9002-0 16096682

[B51] Liu L. Li J. Liu P. Zhao F. Z. Gao B. Q. Du Y. (2012). A genetic linkage map of swimming crab (*Portunus trituberculatus*) based on SSR and AFLP markers. Aquaculture 344-349, 66–81. 10.1016/j.aquaculture.2012.01.034

[B52] Liu P. Wang L. Wong S. M. Yue G. H. (2016). Fine mapping QTL for resistance to VNN disease using a high-density linkage map in Asian seabass. Sci. Rep. 6, 32122. 10.1038/srep32122 27555039 PMC4995370

[B53] Liu Z. F. Wang X. N. Ma A. J. Zhu L. G. Chang H. W. Sun Z. B. (2022). Construction of a high-density genetic linkage map and QTL mapping of growth and cold tolerance traits in tiger puffer Takifugu rubripes. Aquaculture 561, 738613. 10.1016/j.aquaculture.2022.738613

[B54] Loukovitis D. Sarropoulou E. Batargias C. Apostolidis A. P. Kotoulas G. Tsigenopoulos C. S. (2012). Quantitative trait loci for body growth and sex determination in the hermaphrodite teleost fish Sparus aurata L. Anim. Genet. 43, 753–759. 10.1111/j.1365-2052.2012.02346.x 22497460

[B55] Marín M. C. Rodríguez J. R. Ferrús A. (2004). Transcription of Drosophila troponin I gene is regulated by two conserved, functionally identical, synergistic elements. Mol. Biol. Cell 15, 1185–1196. 10.1091/mbc.e03-09-0663 14718563 PMC363105

[B56] Massault C. Franch R. Haley C. de Koning D. J. Bovenhuis H. Pellizzari C. (2011). Quantitative trait loci for resistance to fish pasteurellosis in gilthead sea bream (*Sparus aurata*). Anim. Genet. 42, 191–203. 10.1111/j.1365-2052.2010.02110.x 20946317

[B57] McKenna A. Hanna M. Banks E. Sivachenko A. Cibulskis K. Kernytsky A. (2010). The Genome Analysis Toolkit: a MapReduce framework for analyzing next-generation DNA sequencing data. Genome Res. 20, 1297–1303. 10.1101/gr.107524.110 20644199 PMC2928508

[B58] Mizukoshi M. Zhang H. Tan E. Igarashi Y. Suzuki Y. Watabe S. (2018). Ultrahigh-density linkage map construction using low-coverage whole-genome sequencing of a doubled haploid population: case study of Torafugu (*Takifugu rubripes*). Genes 9, 120. 10.3390/genes9030120 29495372 PMC5867841

[B59] Moen T. Agresti J. Cnaani A. Moses H. Famula T. Hulata G. (2004). A genome scan of a four-way tilapia cross supports the existence of a quantitative trait locus for cold tolerance on linkage group 23. Aquac. Res. 35, 893–904. 10.1111/j.1365-2109.2004.01082.x

[B60] Moustafa A. Hashemi S. Brar G. Grigull J. Ng S. H. S. Williams D. (2023). The MEF2A transcription factor interactome in cardiomyocytes. Cell Death Dis. 14, 240. 10.1038/s41419-023-05665-8 37019881 PMC10076289

[B61] Mukaka M. M. (2012). Statistics corner: a guide to appropriate use of correlation coefficient in medical research. Malawi Med. J. 24, 69–71. 10.2166/wh.2012.000 23638278 PMC3576830

[B62] Nomura K. Ozaki A. Morishima K. Yoshikawa Y. Tanaka H. Unuma T. (2011). A genetic linkage map of the Japanese eel (*Anguilla japonica*) based on AFLP and microsatellite markers. Aquaculture 310, 329–342. 10.1016/j.aquaculture.2010.11.006

[B63] Ooijen V. JoinMap J. W. (2018). Software for the calculation of genetic linkage maps in experimental populations of Diploid species. Wageningen, Netherlands: Kyazma BV.

[B64] Ottenheijm C. A. Hooijman P. Dechene E. T. Stienen G. J. Beggs A. H. Granzier H. (2010). Altered myofilament function depresses force generation in patients with nebulin-based nemaline myopathy (nem2). J. Struct. Biol. 170, 334–343. 10.1016/j.jsb.2009.11.013 19944167 PMC2856782

[B65] Paaby A. B. Rockman M. V. (2013). The many faces of pleiotropy. Trends Genet. 29, 66–73. 10.1016/j.tig.2012.10.010 23140989 PMC3558540

[B66] Pappas C. T. Krieg P. A. Gregorio C. C. (2010). Nebulin regulates actin filament lengths by a stabilization mechanism. J. Cell Biol. 189, 859–870. 10.1083/jcb.201001043 20498015 PMC2878950

[B67] Potthoff M. J. Olson E. N. (2007). MEF2: a central regulator of diverse developmental programs. Development 134, 4131–4140. 10.1242/dev.008367 17959722

[B68] Purcell S. Neale B. Todd-Brown K. Thomas L. Ferreira M. A. R. Bender D. (2007). PLINK: a tool set for whole-genome association and population-based linkage analyses. Am. J. Hum. Genet. 81, 559–575. 10.1086/519795 17701901 PMC1950838

[B69] Reuter J. A. Spacek D. V. Snyder M. P. (2015). High-throughput sequencing technologies. Mol. Cell 58, 586–597. 10.1016/j.molcel.2015.05.004 26000844 PMC4494749

[B70] Rhode C. Jackson T. K. le Cordeur N. S. Jenkins S. F. Sampson J. E. Vervalle J. (2023). Performance, heritability, and candidate genes for growth in dusky kob (*Argyrosomus japonicus*): implications for genetic improvement during early phase domestication. Aquaculture 577, 739971. 10.1016/j.aquaculture.2023.739971

[B71] Rolland Y. Marighetti P. Malinverno C. Confalonieri S. Luise C. Ducano N. (2014). The CDC42-interacting protein 4 controls Epithelial cell Cohesion and Tumor dissemination. Dev. Cell 30, 553–568. 10.1016/j.devcel.2014.08.006 25203208

[B72] Schmidt E. M. Kraemer B. F. Borst O. Munzer P. Schonberger T. Schmidt C. (2012). SGK1 sensitivity of platelet migration. Cell. Physiol. biochem. 30, 259–268. 10.1159/000339062 22759972

[B73] Silva L. C. Wang S. Zeng Z. (2012). Composite interval mapping and multiple interval mapping: procedures and guidelines for using windows QTL cartographer. Methods Mol. Biol. 871, 75–119. 10.1007/978-1-61779-785-9_6 22565834

[B74] Sun C. Niu Y. Ye X. Dong J. Hu W. Zeng Q. (2017). Construction of a high-density linkage map and mapping of sex determination and growth-related loci in the Mandarin fish (*Siniperca chuatsi*). BMC Genom. 18, 446. 10.1186/s12864-017-3830-3 PMC5461734 28587594

[B75] Tang H. Z. Liu J. C. Wang Z. R. Zhang L. J. Yang M. Huang J. (2023). Genome-wide association study (GWAS) analysis of black color trait in the leopard coral grouper (*Plectropomus leopardus*) using whole genome resequencing. Comp. Biochem. Phys. D. 48, 101138. 10.1016/j.cbd.2023.101138 37683359

[B76] Tong J. Sun X. (2015). Genetic and genomic analyses for economically important traits and their applications in molecular breeding of cultured fish. Sci. China Life Sci. 58, 178–186. 10.1007/s11427-015-4804-9 25614028

[B77] Vance J. E. (2015). Phospholipid synthesis and transport in mammalian cells. Traffic 16, 1–18. 10.1111/tra.12230 25243850

[B78] Vincent T. C. Patrizia C. B. (2006). Histone deacetylase 1 is essential for oligodendrocyte specification in the zebrafish CNS. Mech. Dev. 123, 24–30. 10.1016/j.mod.2005.10.005 16324829

[B79] Vinod K. (2011). Kosambi and the genetic mapping function. Resonance 16, 540–550. 10.1007/s12045-011-0060-x

[B80] Voorrips R. E. (2002). MapChart: software for the graphical presentation of linkage maps and QTLs. J. Hered. 93, 77–78. 10.1093/jhered/93.1.77 12011185

[B81] Waldegger S. Barth P. Raber G. Lang F. (1997). Cloning and characterization of a putative human serine/threonine protein kinase transcriptionally modified during anisotonic and isotonic alterations of cell volume. Proc. Natl. Acad. Sci. U. S. A. 94, 4440–4445. 10.1073/pnas.94.9.4440 9114008 PMC20741

[B82] Walsh B. Blows M. W. (2011). Abundant genetic variation + strong selection = multivariate genetic constraints: a geometric view of adaptation. Annu. Rev. Ecol. Evol. S. 42, 41–59. 10.1146/annurev.ecolsys.110308.120232

[B83] Wang W. Hu Y. Ma Y. Xu L. Guan J. Kong J. (2015). High-density genetic linkage mapping in turbot (*Scophthalmus maximus* L.) based on SNP markers and major sex- and growth-related regions detection. PLoS One 10, e0120410. 10.1371/journal.pone.0120410 25775256 PMC4361591

[B84] Wang J. Li L. Zhang G. (2016). A high-density SNP genetic linkage map and QTL analysis of growth-related traits in a hybrid family of oysters (Crassostrea gigas × Crassostrea angulata) using genotyping-by-sequencing. G3-Genes Genom. Genet. 6, 1417–1426. 10.1534/g3.116.026971 PMC4856092 26994291

[B85] Wang L. Bai B. Huang S. Liu P. Wan Z. Y. Ye B. (2017). QTL mapping for resistance to Iridovirus in Asian seabass using genotyping-by-sequencing. Mar. Biotechnol. 19, 517–527. 10.1007/s10126-017-9770-8 28758171

[B86] Wang F. K. F. K. Zhu P. C. Zhang X. T. Yu K. Wang C. D. Liu B. (2025). QTL mapping and candidate gene identification for lower temperature tolerance and growth traits using whole genome re-sequencing in *Argopecten scallops* . Aquaculture 595, 741513. 10.1016/j.aquaculture.2024.741513

[B87] Wang X. X. Zhang G. Gao D. Ge Y. Cheng Y. Wang X. (2025). Whole-genome sequencing reveals the progress of genetic breeding in *Eriocheir sinensis* . Animals 15, 77. 10.3390/ani15010077 39795020 PMC11718898

[B88] Wang S. L. S. L. Luo L. F. Yu Y. Fu Y. Y. Gao Z. X. (2025). Whole-genome re-sequencing reveals genetic structure and selection signals of different populations in *Megalobrama amblycephala* . Aquaculture 595, 741548. 10.1016/j.aquaculture.2024.741548

[B89] Webster M. K. Goya L. Ge Y. Maiyar A. C. Firestone G. L. (1993). Characterization of sgk, a novel member of the serine/threonine protein kinase gene family which is transcriptionally induced by glucocorticoids and serum. Mol. Cell. Biol. 13, 2031–2040. 10.1128/mcb.13.4.2031 8455596 PMC359524

[B90] Wu L. N. Yang Y. Wang X. Weng Z. Y. Hua S. J. Li D. (2023). Genome-wide QTL mapping and RNA-seq reveal the genetic variation influencing growth traits in giant grouper (*Epinephelus lanceolatus*). Aquaculture 563, 738944. 10.1016/j.aquaculture.2022.738944

[B91] Yin Y. An W. Zhao J. H. Li Y. L. Fan Y. F. Chen J. H. (2022). Constructing the wolfberry (Lycium spp.) genetic linkage map using AFLP and SSR markers. J. Integr. Agr. 21, 131–138.

[B92] You X. Shan X. Shi Q. (2020). Research advances in the genomics and applications for molecular breeding of aquaculture animals. Aquaculture 526, 735357. 10.1016/j.aquaculture.2020.735357

[B93] Yu Y. Fu J. Xu Y. Zhang J. Ren F. Zhao H. (2018). Genome re-sequencing reveals the evolutionary history of peach fruit edibility. Nat. Commun. 9, 5404. 10.1038/s41467-018-07744-3 30573726 PMC6302090

[B94] Zhang F. Chen S. M. Chen F. D. Fang W. M. Li F. T. (2010). A preliminary genetic linkage map of chrysanthemum (*Chrysanthemum morifolium*) cultivars using RAPD, ISSR and AFLP markers. Sci. Hortic. 125, 422–428.

[B95] Zhang W. W. Wen X. Fan X. Liang Y. S. Li Y. Q. Chen S. L. (2022). Construction of a high-density genetic linkage map and QTL mapping for growth traits in gynogenetic brown-marbled grouper (*Epinephelus fuscoguttatus*). Aquaculture 561, 738710. 10.1016/j.aquaculture.2022.738710

[B96] Zhao M. Liu Y. Bao M. Kato Y. Han J. Eaton J. W. (2002). Vascular smooth muscle cell proliferation requires both p38 and BMK1 MAP kinases. Arch. Biochem. Biophys. 400, 199–207. 10.1016/S0003-9861(02)00028-0 12054430

[B97] Zhu S. R. Zhu Y. A. Meng Q. L. An L. Yu Z. H. Zhang Y. Y. (2019). A preliminary study on karyotype of *Barbus capito* . Chin. Agri. Sci. Bull. 35, 142–145.

[B98] Zhu C. C. Liu H. Pan Z. Chang G. Wang H. Wu N. (2019). Construction of a high-density genetic linkage map and QTL mapping for growth traits in *Pseudobagrus ussuriensis* . Aquaculture 511, 734213. 10.1016/j.aquaculture.2019.734213

