# Supplementary figures and images for: High-density SNP-based linkage map construction and QTL analysis for growth-related traits in Luciobarbus brachycephalus using whole-genome resequencing data
Source: Front Genet. 2025 Aug 18;16:1644874. doi: 10.3389/fgene.2025.1644874 (PMC12399722; doi:10.3389/fgene.2025.1644874)

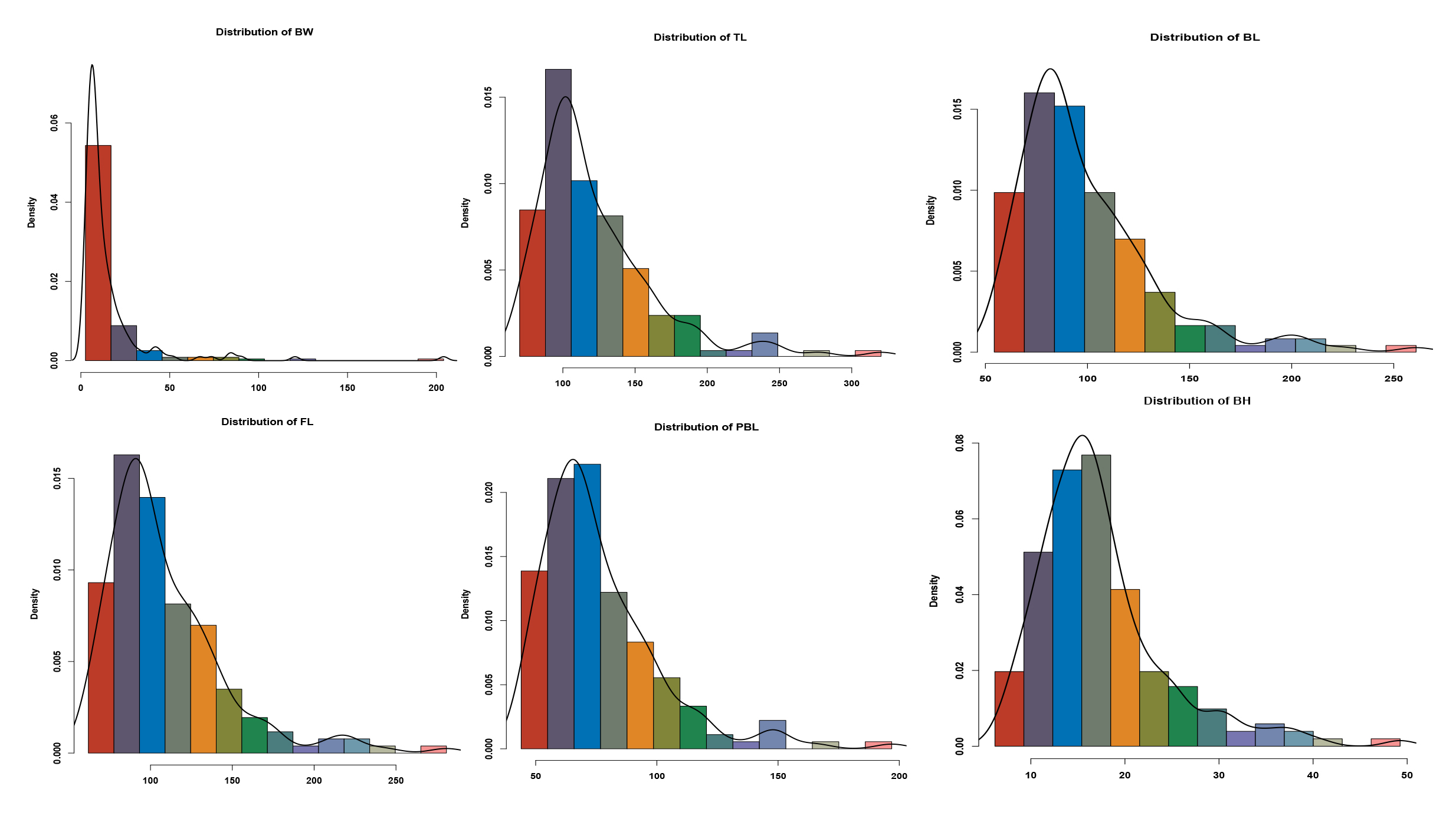

Supplement: Supplementary file 1 [file Image1.jpeg]
